# Predictive microbial feature analysis in patients with depression after acute ischemic stroke

**DOI:** 10.3389/fnagi.2023.1116065

**Published:** 2023-03-23

**Authors:** Shanshan Yao, Huijia Xie, Ya Wang, Nan Shen, Qionglei Chen, Yiting Zhao, Qilu Gu, Junmei Zhang, Jiaming Liu, Jing Sun, Qiuling Tong

**Affiliations:** ^1^Department of Geriatrics, The Second Affiliated Hospital of Wenzhou Medical University, Wenzhou, Zhejiang, China; ^2^Department of Preventive Medicine, School of Public Health and Management, Wenzhou Medical University, Wenzhou, Zhejiang, China; ^3^Department of Neurology, The First Affiliated Hospital of Wenzhou Medical University, Wenzhou, Zhejiang, China

**Keywords:** 16s rRNA, stroke, gut microbiota, depression, microbial signatures

## Abstract

**Introduction:**

Post-stroke depression (PSD) is the most common emotional problem following a stroke, which requires early diagnosis to improve the prognosis. Gut microbiota plays important role in the pathological mechanisms of acute ischemic stroke and influences the outcome of patients. However, the relationship between PSD and gut microbiota remains unknown. Here, we explored whether the microbial signatures of gut microbiota in the patients with stroke could be an appropriate predictor of PSD.

**Methods:**

Fecal samples were collected from 232 acute ischemic stroke patients and determined by 16s rRNA sequencing. All patients then received 17-Hamilton Depression Rating Scale (HAMD-17) assessment 3 months after discharge, and were further divided into PSD group and non-PSD group. We analyzed the differences of gut microbiota between these groups. To identify gut microbial biomarkers, we then established microbial biomarker model.

**Results:**

Our results showed that the composition of gut microbiota in the PSD patients differed significantly from that in non-PSD patients. The genus *Streptococcus*, *Akkermansia*, and *Barnesiella* were significantly increased in PSD patients compared to non-PSD, while the genus *Escherichia-Shigella, Butyricicoccus*, and *Holdemanella* were significantly decreased. Correlation analyses displayed that *Akkermansia, Barnesiella*, and *Pyramidobacter* were positively correlated with HAMD score, while *Holdemanella* was negatively correlated with HAMD score. The optimal microbial markers were determined, and the combination achieved an area under the curve (AUC) value of 0.705 to distinguish PSD from non-PSD.

**Conclusions:**

Our findings suggest that PSD patients had distinct gut microbiota compared to non-PSD patients, and explore the potential of microbial markers, which might provide clinical decision-making in PSD.

## 1. Introduction

Post-stroke depression (PSD) is the most common emotional disorder after stroke (GBD 2015 Neurological Disorders Collaborator Group, 2017), which can increase the risk of mortality and stroke recurrence (Hackett et al., 2014; Cai et al., 2019). About 31% of acute ischemic stroke (AIS) patients may suffer from PSD within 5 years after stroke (Hackett and Pickles, 2014). A meta-analysis showed that delayed diagnosis and treatment of PSD may lead to stroke recurrence and suicide (Cai et al., 2019). Early recognition of patients who were prone to develop PSD was crucial, especially at clinical admission. Close monitoring and early intervention may prevent the adverse outcome (Maaijwee et al., 2016; Baccaro et al., 2019; Fantu et al., 2022; Ladwig et al., 2022). A series of studies evaluated the prediction potential of a variety of scoring systems for PSD, but none were robust enough. Thus, more efficient methods for prediction of PSD were needed.

Risk factors of PSD were multifactorial and complex, including the severity and location of the stroke, social support and personal factors such as gender, age and medical history (Elias et al., 2022; Li et al., 2022; Zhang et al., 2023). Recently, numerous studies revealed the potential association between changes in gut microbiota and prognosis after acute ischemic stroke (Tian et al., 2022; Wang et al., 2022). Previous studies showed that oral administration of antibiotics could reduce neurological impairment and the cerebral infarct volume, relieve cerebral edemas by altering the gut microbiota, while probiotic administration could ameliorate patients’ mood though the regulation of gut microbiota (Chen et al., 2019; Liu et al., 2020). Meanwhile, changes of gut microbiota could also be considered as a prognostic factor in acute ischemic stroke (Benakis and Liesz, 2022; Tian et al., 2022). In recent years, microbial marker severs as a non-invasive diagnosis tool for some diseases (Arnoldussen et al., 2017). Gut microbial marker had the potential to predict the development of AIS, and they may be an effective target tool to influence clinical outcome of AIS. Our previous study showed that the microbiota composition of patients with post stroke cognitive impairment (PSCI) was different from that of non-PSCI patients, characterized by an increase in the abundance of *Proteobacteria* (Ling et al., 2020a). Moreover, we also revealed that patients with post-stroke comorbid cognitive impairment and depression (PSCCID) had lower abundance of short-chain fatty acids (SCFAs) producing bacteria compared with non-PSCCID patients, and revealed that the abundance of Gammaproteobacteria and Enterobacteriaceae was negatively correlated with MoCA score (Ling et al., 2020b). These studies inspired the potential role of the microbiota in PSD progression. There was a correlation between gut microbiota and some complications of acute ischemic stroke, but the link between gut microbiota and PSD has not been well described.

To determine the prediction potential of gut microbiota for PSD outcome on admission, we performed a prospective study. In this study, we compared the difference between the gut microbiota of PSD patients and non-PSD patients, and further explored the “key microbial signatures” of gut microbiota of PSD patients, and searched for possible marker for the diagnosis of PSD patients. Further, through in-depth study on the relationship between the characteristics of gut microbiota and their clinical efficacy, we try to find potential biomarkers for clinical diagnosis, providing a new direction for the screening PSD patients on admission.

## 2. Materials and methods

### 2.1. Patients and sample collection

We enrolled 588 AIS patients at Second Affiliated Hospital of Wenzhou Medical University from September 2020 to July 2021. These patients came from Zhejiang Province with a long-term settlement. Patients with AIS diagnosed according to American Heart Association/American Stroke Association were included (Sacco et al., 2013). The inclusion criteria for this study were: ischemic stroke patients within 3 days after onset, aged 40–95 years, without special diets, such as vegetarianism, no communication deficit (e.g., aphasia and deafness). Patients following conditions were excluded: transient ischemic attack or lacunar infarction; depression, anxiety or other mental illnesses diagnosed or psycho-emotional drug used before the onset of stroke; accompanied with severe systemic diseases (renal failure, respiratory failure, and circulatory failure); medical histories of other central nervous system diseases; failure to complete psychological assessment; use antidepressants, antibiotics or probiotics within 3 months. Eighty-one AIS patients were excluded according to the exclusion criteria, 116 AIS patients failed to collect stool samples, 159 AIS patients lost follow-up, 232 AIS patients that could be analyzed (Supplementary Figure 1). The protocol was approved by the Medical Ethics Committee of the Second Affiliated Hospital of Wenzhou Medical University. All clinical features were recorded according to standard procedures. The peripheral venous blood of the participants was measured at admission. We collected stool samples from each AIS patients at admission.

### 2.2. Demographic and clinical data

Demographic and clinical baseline data were collected, such as age, gender, marital status, education level, smoking and alcohol drinking, past disease history, education level, NIHSS scale, MMSE scale, baseline Barthel Index (BI), baseline blood pressure, baseline blood glucose and lipid levels, other lab results, imaging data, severity stratification, complications, and outcomes. The National Institute of Health Stroke Scale (NIHSS) was used to evaluate the degree of neurological impairment. The cognitive level was assessed by Minimum Mental State Examination (MMSE). PSD was diagnosed with a HAMD score ≥8 according to 17-item Hamilton Depression Scale (HAMD-17) for 3 months after AIS onset (Zimmerman et al., 2013).

### 2.3. Fecal sample collection and analysis

All patients provided fresh stool within 1 week of admission. Fecal samples were collected and immediately transferred to the laboratory. The 200 mg feces samples were placed into a 2 ml sterile centrifuge tube and labeled. All specimens were stored at −80°C within 30 min of preparation. DNA of fecal samples was extracted by DNA Kit (Omega Bio-tek, Norcross, GA, USA.) according to the manufacturer. The DNA concentration and purity were determined using NanoDrop 2000 UV-vis spectrophotometer (Thermo Scientific, Wilmington, DE, USA), and quantified by 1% agarose gel. After DNA extraction, the V3-V4 16S ribosomal RNA gene region was amplified with forward primer (5′-ACTCCTACGGGAGGCAGCAG-3′) and the reverse primer (5′-GGACTACHVGGGTWTCTAAT-3′). Sequencing was performed on the Illumina MiSeq platform (Illumina, San Diego, CA, USA). Raw fastq files were quality-filtered by Trimmomatic and merged by FLASH.

Chao1 and Shannon indexes were applied to analyze the alpha diversity of gut microbiota. A Wilcoxon rank sum test was performed to compare the alpha diversity of groups. Use the rarefaction curves to assess differences in richness between the two groups. The linear discriminant analysis (LDA) effect size (LEfSe) was performed using the Kruskal–Wallis test with LDA score of >2 used as thresholds. Differences in microbiota composition between groups were analyzed using Wilcoxon rank sum test. The Spearman correlation coefficients were calculated and visualized by heatmap. To determine the correlation between the microbial communities in the PSD and Non-PSD group, we constructed correlation network diagram inferred from the abundance of ASV with the threshold of 0.6. Spearman correlation was applied to determine the positive or negative relationship between the ASVs. Nodes were colored by phylum and size was related to the abundance of taxa. ROC curves and the AUC were used to confirm the specificity and sensitivity of the characteristic gut microbiota to the diagnosis of PSD.

### 2.4. Statistical analysis

Data were analyzed by SPSS 22.0 software. Categorical variables were presented as numbers with percentages, and continuous variables were shown as means with standard deviation (SD), or medians with interquartile range (IQR) according to the Kolmogorov-Smirnov normality test. Mann–Whitney U-test was used for continuous variables and the Chi-squared test was used for categorical variables. All variables showing a trend in association in univariate analysis (*P* < 0.10) were included in the multivariable model. The relative risk was expressed as the odds ratio (OR) with a 95% confidence interval (CI). *P* values < 0.05 was considered significant.

## 3. Results

### 3.1. Clinical characteristics of enrolled patients

There were 88 patients diagnosed with PSD (37.93%) after 3-month follow-up. The baseline characteristics of the patients in the two groups are shown in Table 1. In the PSD group, the mean age was 65.43 years old, 46.6% were female, and median education level 2 (2–2). The mean age was 64.57 years old in the non-PSD group, with 29.9% being female and with 2 (2–3) being education level. Univariate analysis showed that the differences between the two groups were gender, education level, diabetes mellitus, alcohol drinking, Barthel Index, FT3, baseline NHISS scores, baseline MMSE scores, and baseline PSQI scores (all *P* < 0.05). Multivariable logistic regression showed that lower MMSE score was an independent risk factors for PSD (*P* = 0.040, OR 0.840, 95% CI 0.711–0.992).

**TABLE 1:** Demographic and clinical characteristics of two groups.

Variables	PSD (*n* = 88)	Non-PSD (*n* = 144)	*P*-value
**Demographic parameters**			
Age (years), mean (SD)	65.43 ± 11.52	64.57 ± 11.81	0.056
Female, *n* (%)	41 (46.6)	43 (29.9)	0.010
Education level, median (IQR)	2 (2–2)	2 (2–3)	0.012
Marriage, *n* (%)	74 (84.1)	131 (91.0)	0.113
**Vascular risk factors**			
Hypertension, *n* (%)	67 (78.4)	107 (74.3)	0.755
Diabetes mellitus, *n* (%)	40 (45.5)	46 (31.9)	0.039
Hyperlipidemia, *n* (%)	52 (59.1)	88 (61.1)	0.294
History of CVD, *n* (%)	17 (19.3)	24 (16.7)	0.607
Smoking, *n* (%)	26 (29.5)	55 (40.5)	0.180
Alcohol drinking, *n* (%)	18 (20.5)	54 (37.5)	0.006
SBP (mmHg), mean (SD)	153.77 ± 23.95	154.07 ± 19.53	0.963
DBP (mmHg), median (IQR)	85 (76–99)	89 (76–96.25)	0.061
**Clinical characteristics**			
Right side lesion location, *n* (%)	46 (52.3)	69 (47.9)	0.520
BI at discharge, median (IQR)	75 (60–95)	95 (65–100)	0.001
NIHSS, median (IQR)	2 (1–3)	2 (1–4)	0.015
MMSE, median (IQR)	24 (20–26)	25 (22–27.25)	0.005
**Laboratory index**			
Hs-CRP (mg/L), median (IQR)	1.54 (0.60–3.46)	1.21 (0.71–2.86)	0.517
Folic acid (ng/ml), median (IQR)	8.79 (7.21–12.49)	9.41 (7.21–11.91)	0.798
Vitamin B12 (pg/ml), median (IQR)	315 (237–601)	321 (123.25–408.75)	0.253
Uric acid (μmol/L), median (IQR)	300 (241.5–337.5)	317 (256.75–374.5)	0.065
TSH (μIU/ml), median (IQR)	2.18 (1.39–3.12)	1.77 (1.18–2.73)	0.744
FT3 (pmol/ml), median (IQR)	2.96 ± 0.39	3.05 ± 0.37	0.029
FT4 (pmol/L), mean (SD)	1.20 ± 0.17	1.17 ± 0.16	0.260

CVD, cerebrovascular disease; HAMD, Hamilton Depression Scale; IQR, interquartile range; MMSE, Mini-mental State Examination; BI, Barthel Index; NIHSS, National Institute of Health Stroke Scale; PSD, post-stroke depression; PSQI, Pittsburgh Sleep Quality Index; SD, standard deviation.

### 3.2. Diversity and distribution of gut microbiota in PSD and non-PSD groups

**FIGURE 1:**
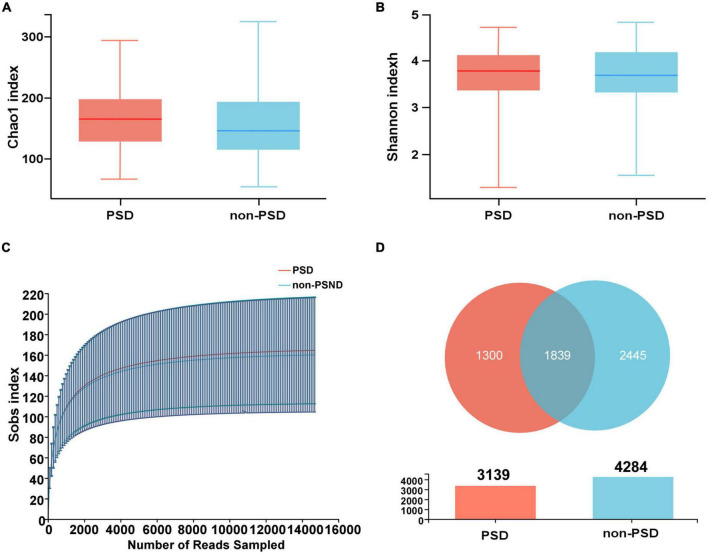
Distribution of gut microbiota in PSD and non-PSD groups. **(A)** Chao1 indexes of gut microbiota α diversity in PSD and non-PSD groups. **(B)** Shannon indexes of gut microbiota α diversity in PSD and non-PSD groups. **(C)** A refraction curve demonstrating richness. **(D)** Venn diagram of gene numbers in PSD and non-PSD groups.

The alpha diversity of the bacterial community was assessed by Shannon and Chao1 indexes. There was no significant difference between PSD and non-PSD groups (*P* = 0.9029, 0.3832), indicating similarity in community richness and diversity between PSD and non-PSD patients (Figures 1A, B). The rarefaction curves (Figure 1C) showed that the richness of PSD group tended to be higher than that of the non-PSD group. The Venn diagram (Figure 1D) showed the common and unique ASVs detected in PSD and non-PSD groups. The number of shared ASVs was 1,839. The number of unique ASVs in PSD group was 1,300, while that in PSND group was 2,445. As shown in Supplementary Figure 2, the gut microbiota beta-diversity between PSD and non-PSD groups was different.

**FIGURE 2:**
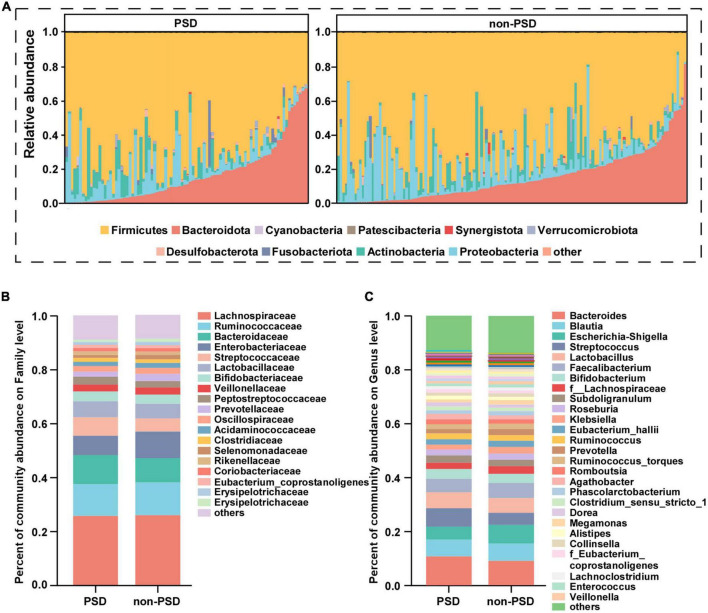
Composition of gut microbiota between PSD and non-PSD groups. **(A)** Predominant bacteria at the phylum level, each bar represents one sample. Relative abundances of bacteria between groups at the family level **(B)** and genus **(C)** level.

**FIGURE 3:**
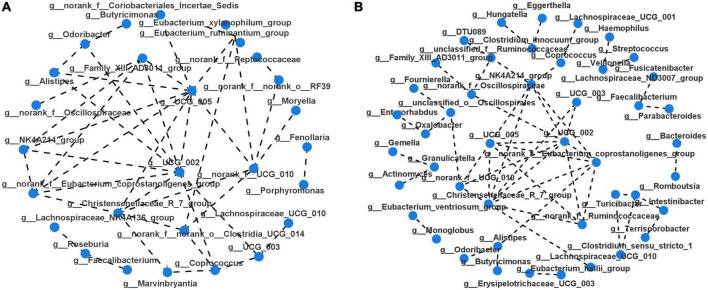
Microbiota correlation network of PSD and non-PSD groups. Each circular node represents a genus. The connecting lines between the two samples represent their Spearman index correlation. **(A)** Correlation network of non-PSD. **(B)** Correlation network of PSD.

Taxonomic profiles at different levels of two groups were presented in Figure 2. phylum Firmicutes, Bacteroidota, Proteobacteria, and Actinobacteria were the predominant in two groups (Figure 2A). Taxonomic classification at the family level showed that most of the intestinal bacteria detected fell into Lachnospiraceae, Ruminococcaceae, Bacteroidaceae, Enterobacteriaceae, Streptococcaceae, Lactobacillaceae, Bifidobacteriaceae, Veillonellaceae, Peptostreptococcaceae, Prevotellaceae, and Oscillospiraceae, which occupied >80% of the total microbiota in PSD group (Figure 2B). Compared with the non-PSD group, PSD group showed lower relative abundances of Enterobacteriaceae and higher relative abundance of Bacteroidacea and Streptococcaceae. At the genera level (Figure 2C), the top five enriched genera were *Bacteroides, Blautia, Escherichia-Shigella, Streptococcus*, and *Lactobacillus*. PSD group showed lower relative abundances of *Escherichia-Shigella* and higher relative abundance of *Bacteroides and Streptococcus* compared with the non-PSD group. Co-occurrence clustering analysis of the top 200 genera of relative abundance were presented in Figure 3. The absolute value of coefficient was set as more than 0.6. The network of PSD contained a total of 45 nodes and 50 edges with the average degree of 2.22 and average clustering of 0.16. The network of non-PSD contained a total of 27 nodes and 42 edges with the average degree of 3.03 and average clustering of 0.21. The results showed that the microbial networks in PSD were sparser than in non-PSD. The clustering coefficient of gut microbiota network in PSD group was lower than that in non-PSD group, indicating that the compactness of the network was reduced (from 6 to 5).

### 3.3. Differential signatures of gut microbiota in PSD and non-PSD groups

**FIGURE 4:**
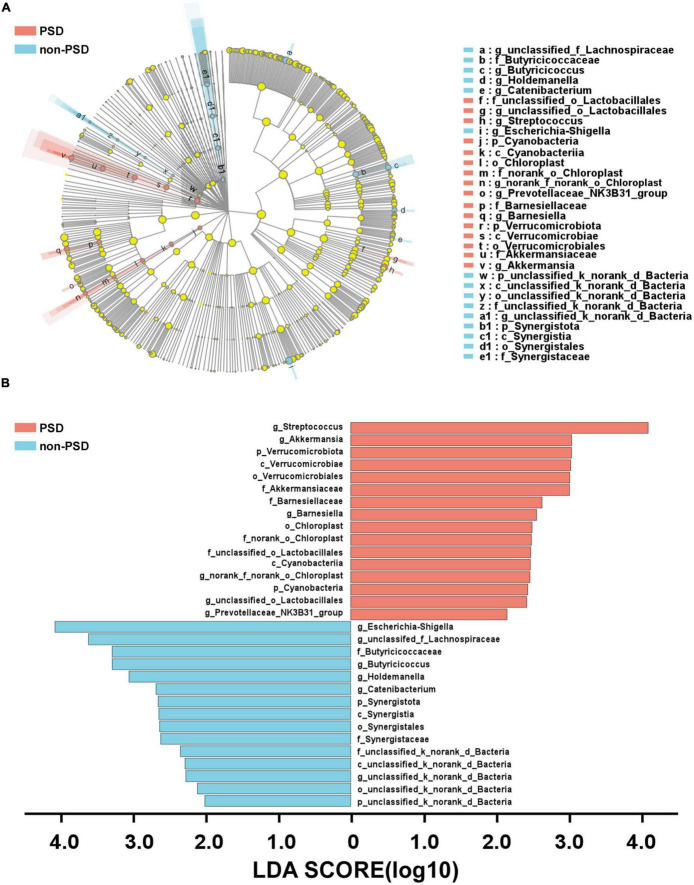
Differential bacteria of gut microbiota between PSD and non-PSD groups. **(A)** Annotated branch diagram of the different bacteria between PSD and non-PSD patients. **(B)** LEfSe analysis showing the most differentially abundant taxa between PSD and non-PSD groups. Red and green bars represented species with relatively higher abundance in PSD and non-PSD patients, respectively. bacterial taxa are shown with a LDA score >2.0.

**FIGURE 5:**
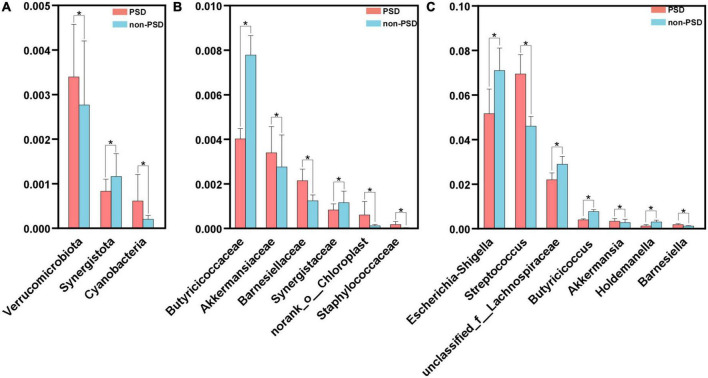
Comparison of the significant bacteria in PSD and non-PSD groups. The relative abundances of the significant bacteria at the phylum level **(A)**, the family **(B)** and the genus level **(C)** in PSD patients compared with non-PSD patients. *P < 0.05.

A LEfSe analysis was used to screen the differential biomarkers of the gut microbiota to explore the specific bacteria associated with PSD. The taxonomic cladogram showed the dominant species of gut microbiota in the two groups from phylum level to genus level (Figure 4A). We marked 31 distinguishing taxa with differential abundances between two groups by LDA scores above 2.0 (Figure 4B). Compared with the non-PSD group, the abundance of phylum Verrucomicrobiota (*P* = 0.048) and Cyanobacteria (*P* = 0.011) in PSD group is higher, and the abundance of phylum Synergistota (*P* = 0.014) is lower (Figure 5A, all *P* < 0.05). Compared with the non-PSD group, the abundance of family Akkermansiaceae (*P* = 0.035), Barnesiellaceae (*P* = 0.0016), Norank_o_Chloroplast (*P* = 0.035) and Staphylococaceae (*P* = 0.0015) in PSD group is higher, and the abundance of family Butyricicoccaceae (*P* = 0.0099) and Synergistaceae (*P* = 0.014) is lower (Figure 5B). At the genus level, PSD exhibited a lower relative abundance of *Escherichia-Shigella* (*P* = 0.043), *unclassified_f_Lachnospiraceae* (*P* = 0.039), *Butyricicoccus* (*P* = 0.012), and *Holdemanella* (*P* = 0.025), and a higher relative abundance of *Streptococcus* (*P* = 0.042), *Akkermansia* (*P* = 0.035), and *Barnesiella* (*P* = 0.0043) (Figure 5C).

### 3.4. Correlation analysis of gut microbiota composition and clinical indicators

**FIGURE 6:**
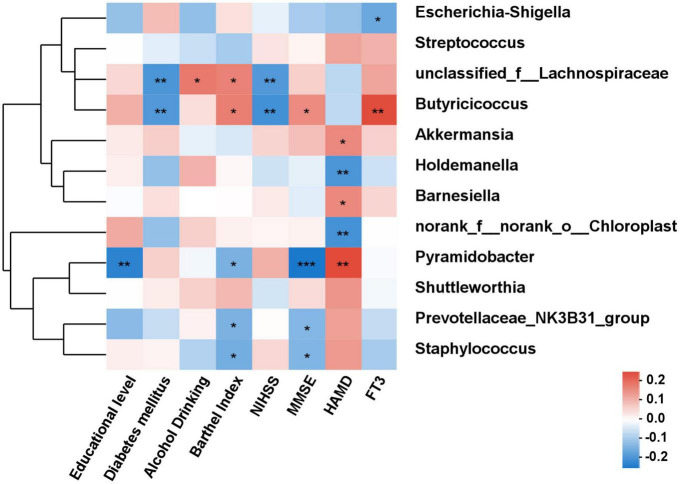
The correlation analysis between the differential bacteria and clinical indexes. Spearman correlations between gut microbiota and clinical indicators. Red and blue indicate positive and negative correlations, respectively. *P < 0.05, **P < 0.01, ***P < 0.001.

As shown in Figure 6, the correlations between differential species and diverse clinical indexes were estimated by the Spearman correlation analysis. The clinical indexes were mainly involving demographic characteristics (educational level, diabetes mellitus, alcohol drinking and Barthel index), laboratory examinations (FT3) and psychiatric outcomes evaluation (NIHSS and MMSE). *Akkermansia* (*P* < 0.05), *Barnesiella* (*P* < 0.05), and *Pyramidobacter* (*P* < 0.01) were positively related with HAMD, while *Holdemanella* (*P* < 0.01) and *norank_f_norank_o_Chloroplast* (*P* < 0.01) negatively connected with HAMD. MMSE score was positively associated with *Butyricicoccus* (*P* < 0.05), while negatively associated with *Pyramidobacter* (*P* < 0.001).

### 3.5. Diagnostic potential of PSD based on gut microbial markers

**FIGURE 7:**
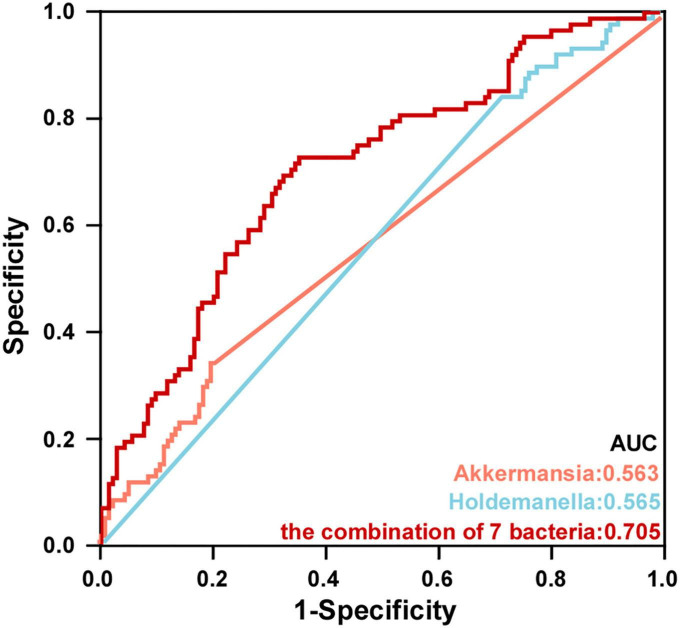
Potential microbiota biomarkers distinguished PSD group from non-PSD group. ROC curve showed the ability to differentiate PSD group from non-PSD group based on specific bacteria.

To further explore the value of microbial signatures in the prediction of PSD, this study picked out genera *Akkermansia* and *Holdemanella* which were closely related to HAMD with relatively high abundance, and the top seven bacteria. Then, receiver operating characteristic (ROC) curve analyses were carried out according to the relative abundance of the top seven bacteria. As shown in Figure 7, *Akkermansia*, *Holdemanella* and the combination of seven bacteria in the distinguishing between PSD and non-PSD had AUC of 0.563, 0.565, and 0.705, respectively (95% CI: 0.486–0.640;95% CI: 0.491–0.640;95% CI: 0.634–0.771), suggesting that the characteristic bacteria might be a well candidate for the diagnosis of PSD.

## 4. Discussion

The pathogenesis of PSD was usually affected by many factors, including gut microbiota. Our analysis revealed a significant difference in gut microbiota composition between PSD and non-PSD groups. Then, we proposed that bacterial biomarkers, such as diversity, abundance feature and specific bacteria, might be useful to identify patients who were likely to develop PSD.

In this study, gender, education level, diabetes mellitus, alcohol drinking, Barthel Index, FT3 differ significantly in PSD and non-PSD, which may be attributed to the occurrence of PSD compared with non-PSD. Many researchers have investigated a series of scoring systems from PSD prediction (Pan et al., 2022; Yi et al., 2022). Prospective studies assessed the predictive power of HAMD score for PSD, which were commonly used in clinical (Kang et al., 2013). There were still flaws in this method. Besides, HAMD score was usually assessed 3 months after the stroke discharge, which is to the disadvantage of early clinical judgment of PSD. However, more relevant data should be included to improve the early recognition of patients.

A large amount of evidence showed that the balance of gut microbiota was disrupted in patients with various brain diseases, such as Parkinson’s disease, Alzheimer’s disease, Wilson’s disease, depression, etc. (Bravo et al., 2011; Dinan and Cryan, 2013; Ait-Belgnaoui et al., 2014; Kennedy et al., 2017). Moreover, several clinical studies have reported that patients with AIS exhibit gut dysbiosis and, in turn, changes in the gut microbiota affected neuroinflammatory and functional outcome after brain injury (Singh et al., 2016). Patients with PSCI also had significantly different gut microbiota at multiple taxonomic levels, compared to AIS patients without cognitive impairment. Further study showed that fecal microbiota transplantation from patients to stroke mice was performed to examine the causal relationship between the gut microbiota and PSCI. These results indicated close contact between gut microbiota and complications after a stroke (Benakis et al., 2016). This study analyzed the characteristics of gut microbiota in patients with PSD. Although there was no significant difference in α diversity of PSD group compared with non-PSD group, but profound alteration in gut microbial structure were discovered in PSD patients. In this study, compared with the non-PSD group, the microbial composition of PSD group was characterized by a decrease in the abundance of the genus *Escherichia-Shigella, Butyricicoccus*, and *Holdemanella* as well as an increase in the abundance of the genus *Streptococcus*, *Akkermansia*, and *Barnesiella.* Moreover, three bacteria *Akkermansia, Barnesiella*, and *Pyramidobacter* were positively correlated with HAMD score, while *Holdemanella* was negatively correlated with HAMD score, which might predict PSD patients. *Akkermansia* is generally considered to be beneficial to human physiology (Kong et al., 2016), however, its changes are inconsistent in many brain diseases. The unpredictable restraint stress potentiated rotenone-induced effects in the colon including increased *Akkermansia* (Dodiya et al., 2018). Studies have shown that the abundance of *Akkermansia* in brain diseases including multiple sclerosis (Cantarel et al., 2015; Berer et al., 2017), PD (Heintz-Buschart et al., 2018), and AD patients (Li et al., 2019) were increased. Our previous studies demonstrated that the abundance of *Akkermansia* in the sepsis-induced cognitive impairment mice (Liu et al., 2021) and AD model mice (Sun et al., 2019) were significantly increased. This contrasts with former studies, where a decrease in the genus *Akkermansia* in PD (Aho et al., 2019) and APP/PS1 mouse model (Harach et al., 2017) were reported. The effect of *Akkermansia* was inconsistent, which has both beneficial effect and negative effect (Desai et al., 2016; Kong et al., 2016). *Akkermansia* was involved in immune regulation, which possessed both regulatory and pro-inflammatory properties (Derrien et al., 2004). *Akkermansia* can induce inflammatory responses, neurotoxicity, and blood-brain barrier disruption in the microenvironment of gliomas through its ability to degrade the intestinal mucosal layer (Patrizz et al., 2020), could aggravate inflammation during infection (Ganesh et al., 2013). *Streptococcus* has been shown to exhibit pro-inflammatory responses. In this study, the genus *Streptococcus* was significantly increased in PSD patients. A case-control study that genera *Streptococcus* were enriched in the depression school-aged children compared with healthy controls (Ling et al., 2022). It was reported that genus *Streptococcus* were significantly increased in the major depressive disorder and bipolar disorder with current major depressive episode groups compared with health participants (Rong et al., 2019). These were characterized by increased relative abundances of taxa that include strains with inflammatory properties, suggesting a potential mediator of inflammation between the gut microbiota and PSD. In addition to exploring the underlying pathogens, this study also evaluated the decreased abundance bacteria in PSD patients, including *Escherichia-Shigella, Butyricicoccus*, and *Holdemanella*. Therefore, bacteria alteration with the increase of opportunistic pathogens and the decrease of beneficial bacteria may be a risk factor for PSD. Moreover, a diagnostic model for PSD was established using seven abundant bacteria, and the AUC area exhibited satisfactory predictive performance. This finding suggested that PSD patients experienced more differences in gut microbial structure related to depressive symptoms, and characteristic bacteria can be used as diagnostic biomarkers for PSD.

There are several limitations to the present study. First, gut microbiota stool samples were collected at a single time point of admission. Future research needs longitudinal design to monitor the diversity and community composition of gut microbiota at different time points, so as to better understand the dynamic changes of microbiota in PSD patients. Second, although we tried to consider the influence of environmental factors (region, diet, educational background) on the depressive state and microbial population of AIS patients, there was still a lack of further detailed and comprehensive information (such as socioeconomic status, social support, seasonal factors, etc.). More detailed dietary and lifestyle factors information of the participants need provided to assess whether and how dietary habits affect the microbial composition of- PSD patients. The participants adopted different diet and style, which may lead to bacterial variations. Lastly, the sample size was limited, thus these conclusions may require careful interpretation. Large-scale multicenter and cross-regional studies are necessary to estimate the role of gut microbiota in predicting the diagnosis of PSD.

In conclusion, this study revealed that gut microbiome of PSD patients has changed, which is a predictor of PSD. *Escherichia-Shigella, Butyricicoccus*, *Akkermansia*, and *Barnesiella* were microbial biomarkers for PSD, which was worthy of further study on clinical application. Our findings may help to early predict PSD and provide information for clinical decision-making of patients.

## Data availability statement

The datasets presented in this study can be found in online repositories. The names of the repository/repositories and accession number(s) can be found below: https://www.ncbi.nlm.nih.gov/, PRJNA905676.

## Ethics statement

This study was approved by Ethics Committee of the Second Affiliated Hospital of Wenzhou Medical University. The patients/participants provided their written informed consent to participate in this study.

## Author contributions

JL, JS, and QT: conception or design of the work. SY, HX, YW, NS, QC, YZ, QG, and JZ: data collection and analysis. All authors contributed to the article and approved the submitted version.

## Supplementary material

Supplementary Figure 1 Flowchart of patients included in the study.

Supplementary Figure 2 Composition of gut microbiota beta-diversity between PSD and non-PSD groups. Beta-diversity illustrating the grouping patterns of PSD and non-PSD groups by principal coordinate analysis.
