# Supplementary figures and images for: Predictive microbial feature analysis in patients with depression after acute ischemic stroke
Source: Front Aging Neurosci. 2023 Mar 23;15:1116065. doi: 10.3389/fnagi.2023.1116065 (PMC10076592; doi:10.3389/fnagi.2023.1116065)

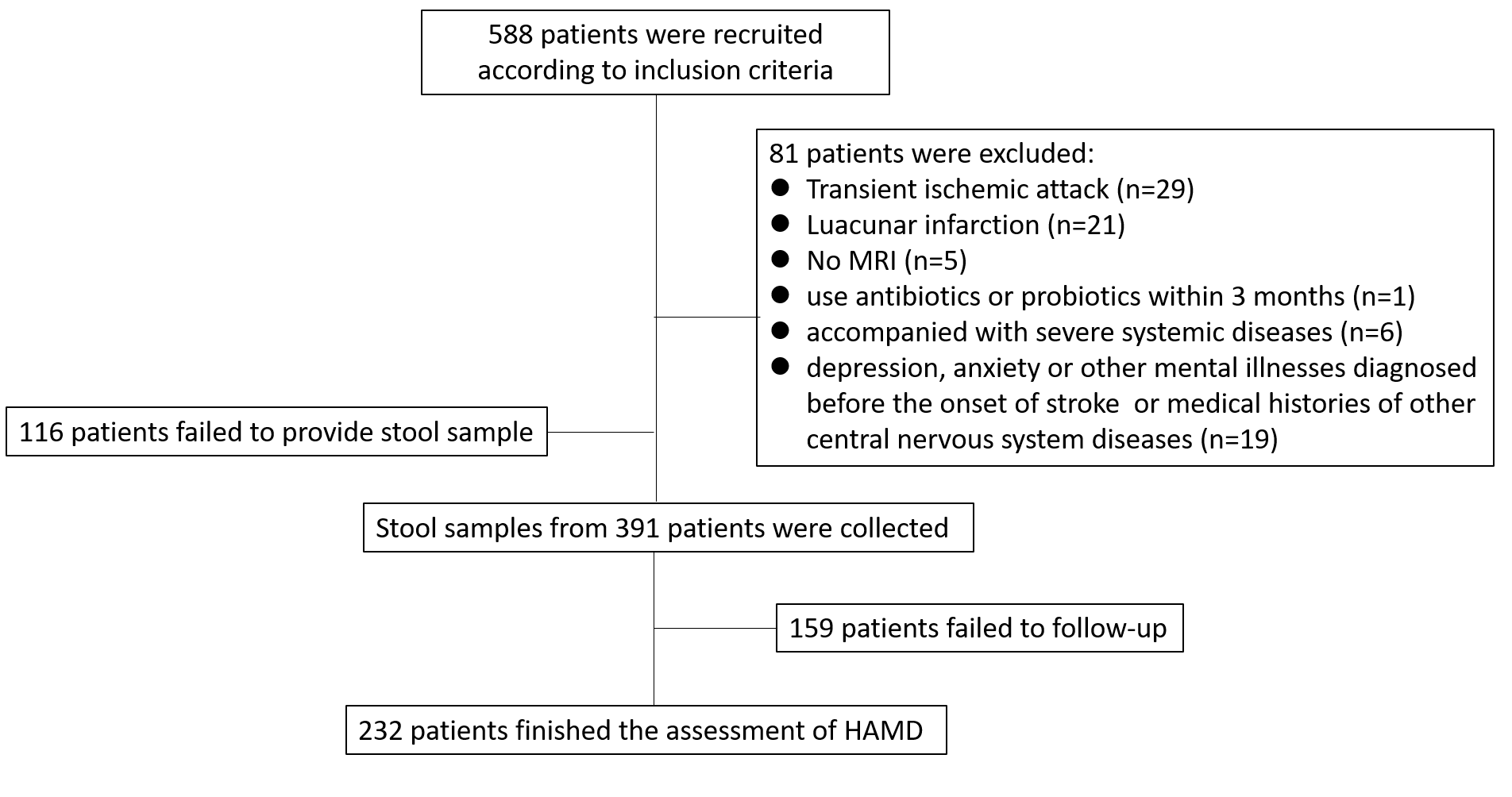

Supplement: Supplementary Figure 1 — Flowchart of patients included in the study. [file Image_1.tif]

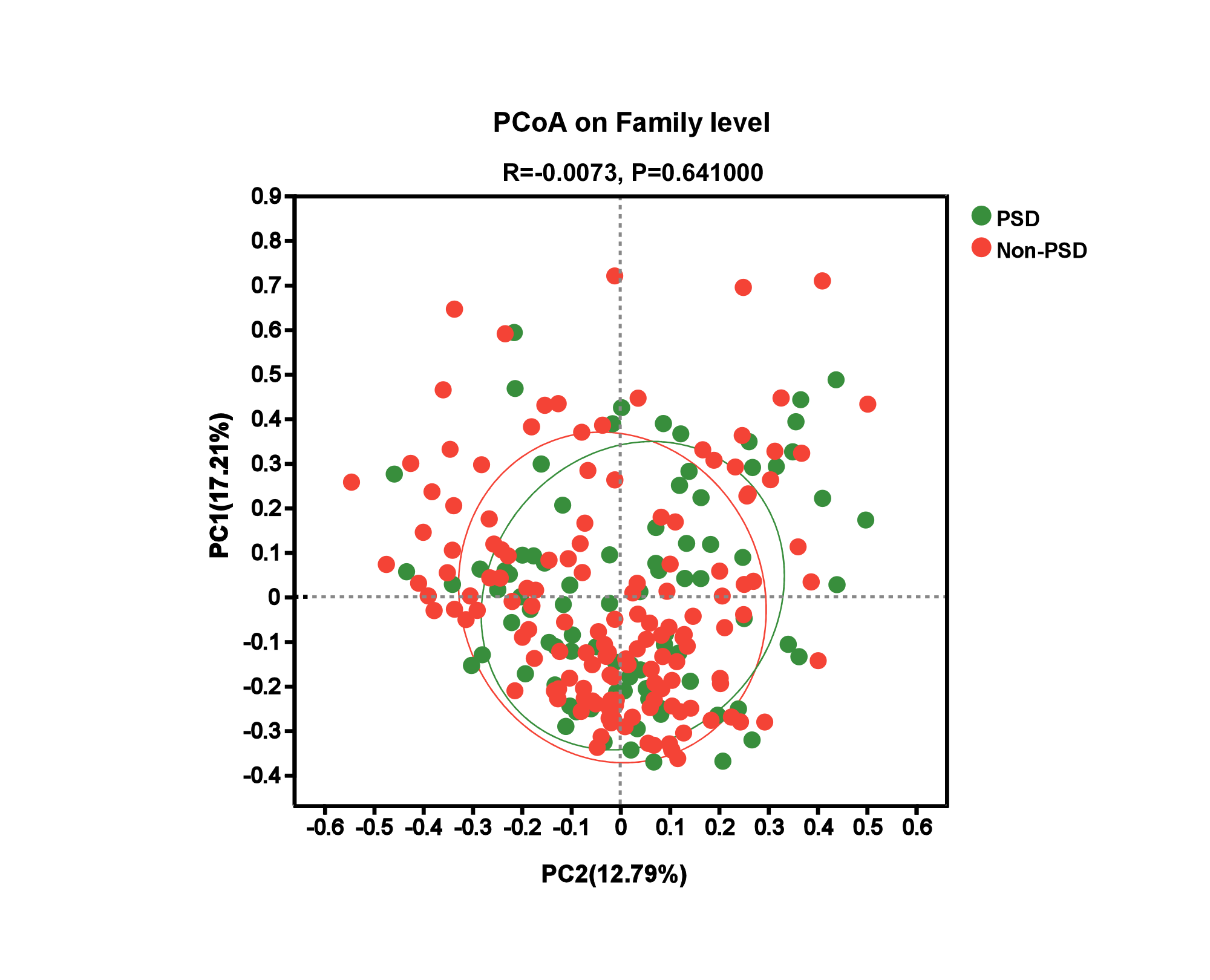

Supplement: Supplementary Figure 2 — Composition of gut microbiota beta-diversity between PSD and non-PSD groups. Beta-diversity illustrating the grouping patterns of PSD and non-PSD groups by principal coordinate analysis. [file Image_2.tif]

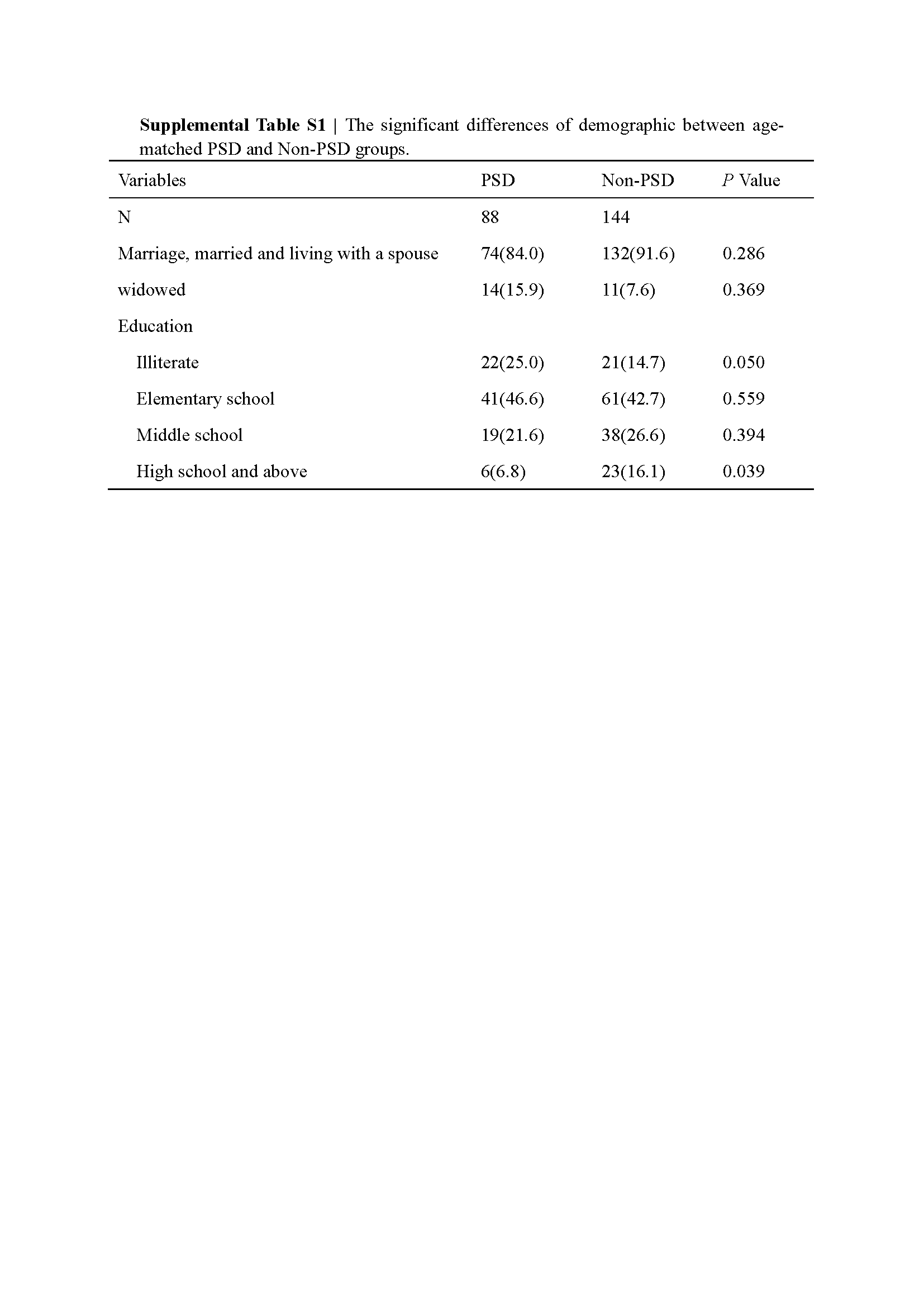

Supplement: Supplementary file 3 [file Table_1.tiff]
